# Response to the Commentary on the paper “The heartbeat evoked potential is a questionable biomarker in nightmare disorder: A replication study. By Bogdany, T., Perakakis, P., Bodizs, R., Simor, P., 2021. NeuroImage Clin. 33, 102933”

**DOI:** 10.1016/j.nicl.2022.103197

**Published:** 2022-09-21

Dear Editors,

We were glad to receive the commentary of Perogamvros et al. on our recent article because we hope that inconsistent findings and different perspectives lead to deeper reflections and new ideas about the relevance of interoception, and more specifically, heartbeat evoked potential (HEP) in nightmare disorder. The authors of the original article argue that in our replication study we failed to replicate the independent variable, namely the clinical sample they compared with control participants. Although we will question this argument, we would like to stress that in our view a replication study is not necessarily an exact copy of the original study, but an attempt to generalise the findings to slightly different populations, especially if the aim is to identify a reliable and robust biomarker.

Our participants were not diagnosed by a clinician, and were not seeking treatment. Perogamvros and colleagues use an analogy between clinical depression and sadness to highlight how their sample of diagnosed patients and our nightmare group might differ. We believe that such an analogy is rather misleading, since sadness (and more generally, negative affect) is a normative human experience (Bonanno et al., 2008), whereas frequent nightmares (>1 per week) characterize only about 2–5 % of the adult population, and are considered to be a risk factor for a variety of mental health complaints (Spoormaker and Montgomery, 2008). Moreover, the authors emphasize that daytime dysfunction related to frequent nightmares is a critical component of nightmare disorder, and take for granted that our sample was free from such daytime complaints. In fact, we examined these daytime dysfunctions by standardized questionnaires assessing affective and cognitive impairments provoked by frequent nightmares, in addition to widely used questionnaires measuring daytime fatigue, sleepiness, sleep quality, daytime anxiety, and ptsd-like symptoms such as intrusive thoughts (Blaskovich et al., 2020, Simor et al., 2012). The results of these questionnaires, as well as the personalized interviews with our participants indicated relatively elevated daytime symptoms and impaired sleep quality related to nocturnal awakenings in the nightmare group; therefore, it is surprising why Perogamvros and colleagues claim that our results reflect emotional homeostasis in healthy participants having (only) bad dreams.

We do not exclude the possibility that the patients in the original study showed more severe symptoms with respect to frequent nightmares; however, the mere fact that the patients in the original study asked for treatment is not sufficient to distinguish the two samples in this regard. For instance, nightmare sufferers rarely seek professional treatment (Schredl, 2010), and unfortunately, targeted treatments for nightmare disorder are practically not available in Hungary (Simor, 2013) (where our sample was selected). Psychometric assessments are able to quantify the severity of frequent nightmares; however, the only data reported by Perogamvros and colleagues are the scores of a depression scale (Perogamvros et al., 2019), that in fact, show comparable values with the scores of our nightmare groups (Blaskovich et al., 2020, Simor et al., 2012).

Examining nightmare sufferers and controls selected from non-clinical groups maximizes the homogeneity of the sample and minimizes the influence of confounding factors. Accordingly, our control participants were selected from the same population, the selection procedure was based on the same psychometric tools, and in our second database we also controlled for the frequency of dream recall. Dream recall frequency is a notoriously overlooked, but crucial variable in nightmare studies, since nightmare sufferers are also characterized by increased dream recall, hence, an appropriate control group should also feature high (but non-dysphoric) dream recall. Unfortunately, it is not clear how the controls were selected in the original study (whether they were selected from the same community, underwent the same interviews, etc.), hindering further speculations about the differences between the original and the replication study.

Perogamvros and colleagues in their letter do not seem to favour the dimensional view of nightmare disorder and argue that daytime distress associated with frequent nightmares distinguishes the pathological and the functional expressions of dysphoric dreaming. In our view, the study of neurocognitive processes in mental health disorders has produced a large amount of valuable data that questions the utility of the categorical approach to diagnose psychiatric conditions, and promotes a dimensional view to better understand the mechanisms of maladaptive and dysfunctional cognitive processes that eventually may lead to severe pathological conditions (McHugh, 2005, Robbins et al., 2012). The identification of potential biomarkers is in line with the dimensional approach, and if such biomarkers prove to be robust and reliable, hopefully they will be integrated into diagnostic and treatment procedures.

Best Regards,

Peter Simor, Ph.D.

Tamás Bogdány, MSc.

Róbert Bódizs, Ph.D.

Pandelis Perkakakis, Ph.D.

## Declaration of Competing Interest

The authors declare that they have no known competing financial interests or personal relationships that could have appeared to influence the work reported in this paper.
